# Tap water as a natural vehicle for microorganisms shaping the human gut microbiome

**DOI:** 10.1111/1462-2920.15988

**Published:** 2022-04-07

**Authors:** Gabriele Andrea Lugli, Giulia Longhi, Leonardo Mancabelli, Giulia Alessandri, Chiara Tarracchini, Federico Fontana, Francesca Turroni, Christian Milani, Douwe van Sinderen, Marco Ventura

**Affiliations:** ^1^ Laboratory of Probiogenomics, Department of Chemistry, Life Sciences, and Environmental Sustainability University of Parma Parma Italy; ^2^ GenProbio Srl Parma Italy; ^3^ Microbiome Research Hub University of Parma Parma Italy; ^4^ APC Microbiome Institute and School of Microbiology, Bioscience Institute, National University of Ireland Cork Ireland

## Abstract

Fresh potable water is an indispensable drink which humans consume daily in substantial amounts. Nonetheless, very little is known about the composition of the microbial community inhabiting drinking water or its impact on our gut microbiota. In the current study, an exhaustive shotgun metagenomics analysis of the tap water microbiome highlighted the occurrence of a highly genetic biodiversity of the microbial communities residing in fresh water and the existence of a conserved core tap water microbiota largely represented by novel microbial species, representing microbial dark matter. Furthermore, genome reconstruction of this microbial dark matter from water samples unveiled homologous sequences present in the faecal microbiome of humans from various geographical locations. Accordingly, investigation of the faecal microbiota content of a subject that daily consumed tap water for 3 years provides proof for horizontal transmission and colonization of water bacteria in the human gut.

## Introduction

Freshwater is estimated to represent about 2.5% of all water on Earth, while the remainder constitutes saltwater from seas and oceans. In developed countries, potable water is readily accessible as tap water and bottled natural mineral water, both of which are subject to strict safety regulations and very regular inspections (Eichler *et al*., 2006). Conventional drinking water treatment plants perform filtration, sedimentation, disinfection and flocculation, thereby allowing assessment of the microbial load (Dodd, 2012; Chao *et al*., 2013; Loubet *et al*., 2016). Nonetheless, some microorganisms may persist and proliferate in drinking water, with bacterial concentrations estimated to be around 10^6^–10^8^ cells per litre (Hammes *et al*., 2008; Hong *et al*., 2010). Thus, like other consumed items, water may represent a natural vehicle of microorganisms able to interact with the human gut and its microbiota (Dimidi *et al*., 2019; Milani *et al*., 2019).

Some studies have observed significant associations between tap water composition and human health (Bouchard *et al*., 2011). In this context, differences were observed in the composition of the gut microbiota of mice that drank water from different sources, including tap water, highlighting an increase of clinically important taxa such as *Acinetobacter* and *Staphylococcus* in the faeces and mucosa‐adhered samples of animals (Dias *et al*., 2018). Similar examples have also been reported in a human population context, given the recent evidence of bacterial spread from microbial biofilms present in drinking water distribution systems (Chan *et al*., 2019) and investigations regarding the potential for tap water to influence human health mediated through the gut microbiota (Bowyer *et al*., 2020).

Microbial populations in drinking water are challenging to assess because most of the bacteria present appear to be non‐culturable (Loy *et al*., 2005; França *et al*., 2015) or are present in a viable‐but‐non‐culturable state (Szewzyk *et al*., 2000). For this reason, metagenomic sequencing approaches represent a well‐established method to study the culturable and unculturable parts of the drinking water microbiota (Brumfield *et al*., 2020; Sala‐Comorera *et al*., 2020). Furthermore, recent studies provide exciting insights into the composition of tap water microbiota by revealing that increased urban development causes shifts in bacterial community composition of water (Simonin *et al*., 2019). Thus, microbial community analyses can be helpful to diagnose environmental conditions as indicators of ecosystem health and water quality condition. In this context, the predominant bacteria that have been detected in drinking water are members of the phyla Actinobacteria and Proteobacteria, with the genera *Afipia*, *Bradyrhizobium* and *Mycobacterium* being prevalent among tap water and drinking fountain samples (Brumfield *et al*., 2020).

In the current study, we investigated the microbiota composition of tap water from the city pipeline of Parma, Italy, using a shallow metagenomics approach, which allows accurate taxonomic profiling of microbial communities from water samples down to species level. In addition, the metagenomics data were used in conjunction with data sets retrieved from the NCBI repository to describe the occurrence of a core tap water microbiota as well as the existence of specific microbial groups that are typical of the various geographical regions investigated. Within this context, we mapped the presence of a microbial taxon shared between the tap water microbiota and the gut microbiota of an individual daily drinking that water, suggesting horizontal transmission by these bacteria, which in turn appears to impact other resident members of the human gut microbiota.

## Results and discussion

### Uncovering tap water microbial biodiversity

**Fig. 1:**
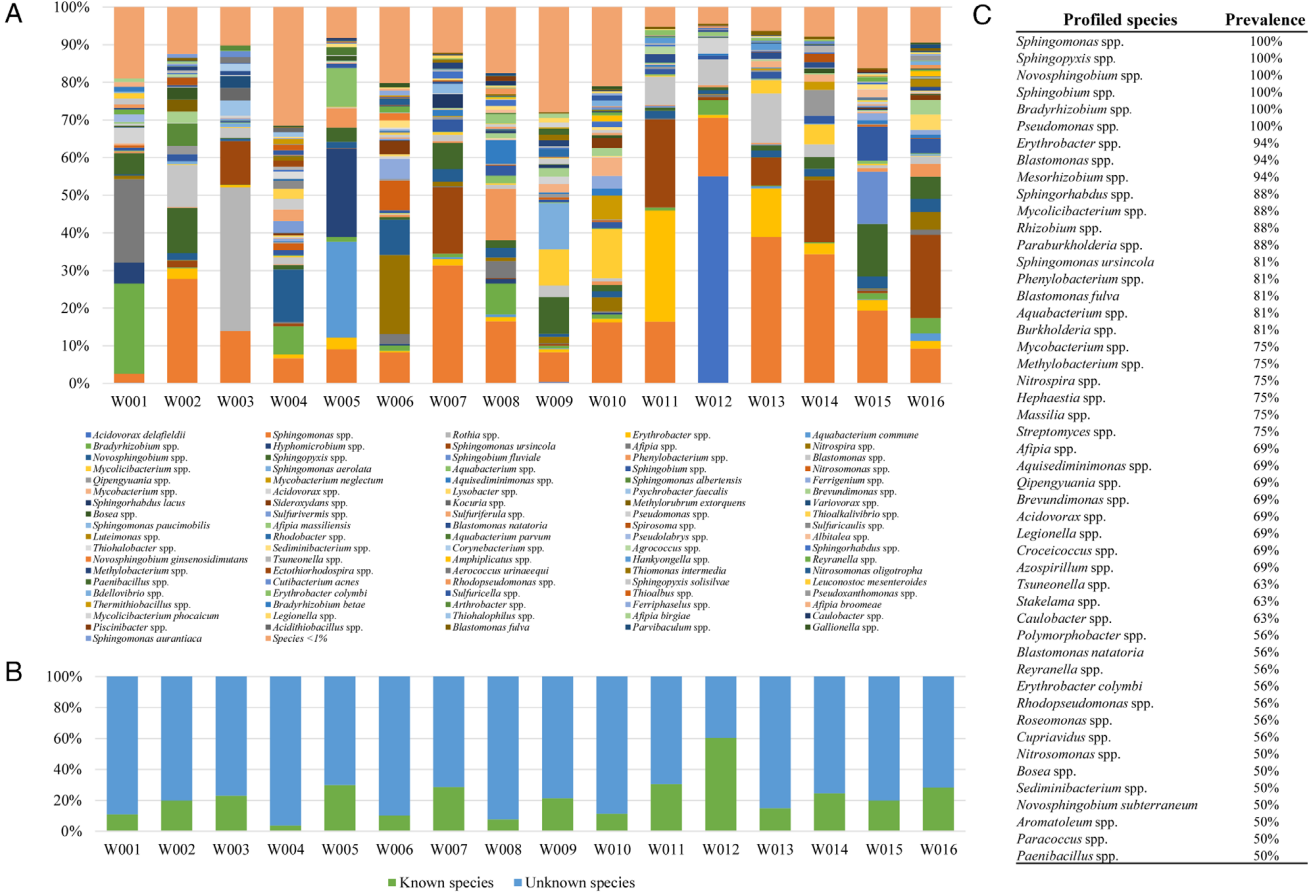
Microbial composition of 16 drinking water samples collected from different locations of the Parma District and delivered by the city water supply system. Panel A displays a histogram with the relative abundance of each microbial species identified in the analysed samples. From left to right, the colour‐coding order of the legend reflects average abundances from largest to smallest, i.e. 55% *Acidovorax delafieldii* in sample W012, 38.9% *Sphingomonas* spp. in sample W013, and so on. Panel B shows the percentage of unknown and known bacterial species profiled, while Panel C depicts the prevalent bacterial taxa identified among all processed samples.

Sixteen water samples, named W001 to W016, were collected from public fountains (*n* = 12) and household taps (*n* = 4) throughout the Parma district in Italy to explore the microbial biodiversity of water samples across the city (Table 1). Shallow shotgun metagenomic sequencing was performed to identify the microorganisms that populate water samples at species level. Sequencing output constituted about one million paired‐end reads, with an average of 64 thousand reads per sample (Table S1), thus allowing accurate assessment of the microbial species inhabiting each water sample (Hillmann *et al*., 2018). This taxonomic survey revealed that just five species were present in particular samples at a relative abundance higher than 10%, i.e. *Acidovorax delafieldii* (55% in W012), *Aquabacterium commune* (26% in W005), *Sphingomonas ursincola* (23% in W011), *Sphingobium fluviale* (14% in W015) and *Sphingomonas aerolata* (12% in W009) (Fig. 1). As reported in the literature, these species had previously been identified in biofilms of drinking water samples (Kalmbach *et al*., 1999; Busse *et al*., 2003; Morohoshi *et al*., 2017). Moreover, unclassified members of 13 additional bacterial genera were identified with relative abundances ranging from 10% to 39%, highlighting the marked presence of as yet to be isolated and characterized bacterial species (Fig. 1). Interestingly, the major difference identified between samples collected from public fountains and household taps was represented by the presence of *Sphingomonas*, found as the most abundant taxa in nine out of 12 public fountains water samples (Table 1).

**Table 1:** Water samples of the Parma district.

Sample name	Sample type	Sampling day	Most abundant species	SRA	Bioproject
W001	Tap water	22/06/2021	*Bradyrhizobium* sp.	SRR18015330	PRJNA806724
W002	Public drinking fountain water	05/05/2021	*Sphingomonas* sp.	SRR18015329	PRJNA806724
W003	Public drinking fountain water	05/05/2021	*Rothia* sp.	SRR18015322	PRJNA806724
W004	Tap water	28/05/2021	*Novosphingobium* sp.	SRR18015321	PRJNA806724
W005	Tap water	06/05/2021	*Aquabacterium commune*	SRR18015320	PRJNA806724
W006	Tap water	23/04/2021	*Nitrospira* sp.	SRR18015319	PRJNA806724
W007	Public drinking fountain water	10/05/2021	*Sphingomonas* sp.	SRR18015318	PRJNA806724
W008	Public drinking fountain water	07/05/2021	*Sphingomonas* sp.	SRR18015317	PRJNA806724
W009	Public drinking fountain water	17/05/2021	*Sphingomonas aerolata*	SRR18015316	PRJNA806724
W010	Public drinking fountain water	23/04/2021	*Sphingomonas* sp.	SRR18015315	PRJNA806724
W011	Public drinking fountain water	17/05/2021	*Erythrobacter* sp.	SRR18015328	PRJNA806724
W012	Public drinking fountain water	06/05/2021	*Acidovorax delafieldii*	SRR18015327	PRJNA806724
W013	Public drinking fountain water	04/05/2021	*Sphingomonas* sp.	SRR18015326	PRJNA806724
W014	Public drinking fountain water	04/05/2021	*Sphingomonas* sp.	SRR18015325	PRJNA806724
W015	Public drinking fountain water	23/04/2021	*Sphingomonas* sp.	SRR18015324	PRJNA806724
W016	Public drinking fountain water	07/05/2021	*Sphingomonas ursincola*	SRR18015323	PRJNA806724

A flow cytometry (FC) assay was employed to enumerate microbial cells present in each water sample, thereby providing information on the absolute number of microbes. Based on the predicted number of colony‐forming units (CFU), analysed samples ranged from 2 × 10^3^ CFU ml^−1^ in W009 to 5.3 × 10^6^ CFU ml^−1^ in W001 (Fig. S1). Interestingly, when excluding sample W001, the average CFU ml^−1^ between water samples was 2.8 × 10^4^, highlighting W001 as an outlier of the analysis with a high level of bacterial cells probably due to downstream effects of the water distribution system (Fig. S1). Then, FC data obtained for each water sample were used to normalize the taxonomically classified reads obtained through shallow shotgun metagenomics according to a previously described method (Lugli *et al*., 2020), thereby allowing an estimation of absolute abundance (Fig. S1). The findings showed a high CFU ml^−1^ number of *Bradyrhizobium* spp. and *Afipia* spp. present in sample W001, and *Rothia* spp. representing a microorganism present at high absolute abundance in sample W003, highlighting bacterial taxa which had also previously been identified in drinking water systems (Brumfield *et al*., 2020).

Furthermore, taxonomic profiling at species level allowed us to identify the core taxonomic elements of the water microbiome, i.e. taxa that occur with the highest prevalence. Notably, in addition to *Sphingomonas ursincola* and *Blastomonas fulva*, which were determined to be the most prevalent species being detected in 13 out of 16 samples, taxonomic profiling also revealed DNA of unknown bacterial species attributable to 30 genera distributed between samples at a prevalence of >70%. Remarkably, these later findings underscore that many members of the microbial drinking water population have as yet not been subject to any characterization (Fig. 1). Among the latter microbial dark matter, unknown members of six genera were identified in all 16 samples, i.e. *Bradyrhizobium*, *Novosphingobium*, *Pseudomonas*, *Sphingobium*, *Sphingomonas* and *Sphingopyxis*. Intriguingly, while *Pseudomonas* is a ubiquitous environmental bacterial genus, members of the other five genera have previously been isolated from groundwater and drinking water (McAlister *et al*., 2002; Yoon *et al*., 2005; Sheu *et al*., 2013; Singh *et al*., 2015; Gulati and Ghosh, 2017). Altogether, these metagenomic data sets highlight the substantial knowledge gap pertaining to drinking water‐associated bacteria.

### Meta‐analysis of the water microbiome across the world

To validate the quality of our metagenomic analysis and compare the taxonomic profiles of water samples identified in the current study with those of other geographical locations, sequencing data of 119 drinking water samples were retrieved from nine metagenomic projects, named P02 to P10, which cover seven different countries, (Table S2). Taxonomic profiling at species level was performed applying the same pipeline and parameters used for samples collected as part of our own study, as reported above. While three samples were discarded due to low sequencing data (<20 000 DNA sequence reads) (Table S1), along with the P04 sample that was not suitable for statistical purposes, the remaining 115 water samples were analysed together with the microbiome data of 16 samples retrieved in the Parma district.

**Fig. 2:**
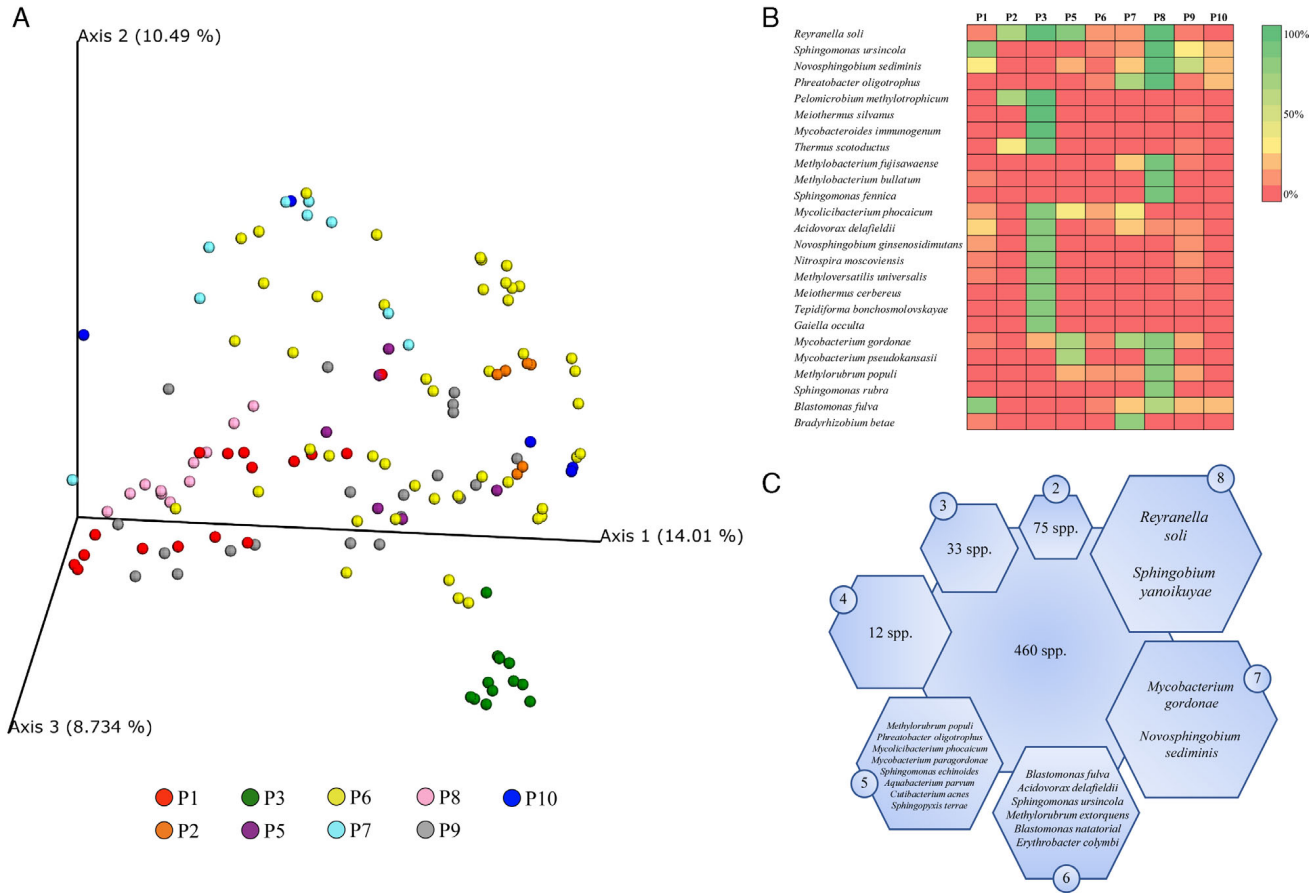
Microbial composition of 132 drinking water samples as obtained from NCBI. Panel A exhibits the PCoA, based on microbial distribution, represented in different colours by means of 10 projects (P1 to P10) using the Bray–Curtis index. Panel B displays a heat map with the prevalence of major microbial players between projects. Panel c reports a schematic overview of the most prevalent bacterial species between projects. Species names and numbers in hexagons represent those taxa prevalent in samples from a number of projects written in circles. P4 data were excluded from the comparisons to avoid bias in the analyses' outcome.

Beta‐diversity investigation represented through principal coordinate analysis (PCoA) based on Bray–Curtis dissimilarity index allowed exploration of the water biome biodiversity as based on different studies (Fig. 2). Marked biodiversity was encountered between almost all projects (PERMANOVA *p*‐value of <0.05) except for P6 (Table S3). These data highlighted unique signatures among water sample microbiomes associated with the same country, possibly due to geographic and environmental factors such as temperature and pH. As similarly reported in P1 (Parma district project), an average of 16% of the microbial DNA of water was classified at species level, while the remaining 84% was attributable to unknown microbial taxa, thus representing a sizable part of the water microbial dark matter. Investigating the prevalence of bacterial taxa between samples of the same project, a limited number of taxa (between 13 and zero) were observed with a prevalence >80%, indicating a high degree of microbial biodiversity and inter‐site variability (Fig. 2). Notably, we included two longitudinal studies (P3 and P8) and observed a higher number of high prevalence taxa (13 and 11 respectively), indicating lower inter‐sample biodiversity, when considering samples collected longitudinally from the same site (Dai *et al*., 2018; Vosloo *et al*., 2021). For example, only *Reyranella soli* was identified with a high prevalence (83%) in P5 samples, while a similar observation was made for *Blastomonas fulva* and *Sphingomonas ursincola* in the case of P1 samples, which showed a prevalence of 81% for these species (Fig. 2).

Interestingly, 84% of unclassified microbial DNA identified among water samples, previously referred as microbial dark matter (Rinke *et al*., 2013), represented the actual core water microbiota (Table 2). In this context, following DNA filtering steps (see Experimental procedures), the microbial portion of a sample that was not classified at species level revealed unknown bacterial species highlighting the absence of a reference genome deposited in the NCBI repository. The five most prevalent microorganisms identified in the 115 drinking water samples have already been identified as major players in the 16 samples collected in the tap water of Parma district, i.e. members of the genera *Bradyrhizobium*, *Sphingomonas*, *Pseudomonas*, *Novosphingobium* and *Sphingobium* (Table 2). Additionally, the DNA of species belonging to *Paraburkholderia*, *Burkholderia* and *Mesorhizobium* was also identified in more than 90% of the profiled waters (Table 2).

**Table 2:** Prevalence of profiled microorganisms between projects.

	P1 (%)	P2 (%)	P3 (%)	P5 (%)	P6 (%)	P7 (%)	P8 (%)	P9 (%)	P10 (%)	All (%)	Core
*Bradyrhizobium* spp.	100	100	100	83	100	100	100	100	100	99	8
*Sphingomonas* spp.	100	100	83	83	100	100	100	100	100	98	7
*Pseudomonas* spp.	100	100	100	83	96	100	100	100	100	98	7
*Novosphingobium* spp.	100	100	83	83	96	100	100	100	100	96	6
*Paraburkholderia* spp.	88	100	92	67	91	100	100	100	100	93	5
*Sphingobium* spp.	100	100	33	83	98	100	100	100	100	92	6
*Burkholderia* spp.	81	100	100	50	89	100	100	100	100	92	6
*Mesorhizobium* spp.	94	100	83	67	85	100	100	95	100	90	4
*Massilia* spp.	75	100	83	67	85	100	100	100	80	88	4
*Cupriavidus* spp.	56	100	100	67	87	78	100	100	100	87	5
*Mycolicibacterium* spp.	88	100	83	67	87	56	82	80	100	83	2
*Methylobacterium* spp.	75	100	25	67	91	100	100	85	100	83	4
*Reyranella* spp.	56	100	100	100	87	100	100	60	100	83	6
*Sphingopyxis* spp.	100	67	58	50	76	100	100	100	60	83	4
*Roseomonas* spp.	56	100	75	50	83	100	100	90	100	83	4
*Rhizobium* spp.	88	100	33	67	78	100	100	100	100	83	5
*Streptomyces* spp.	75	100	100	67	76	100	82	85	80	83	3
*Azospirillum* spp.	69	100	58	50	83	100	100	85	100	82	4
*Aquabacterium* spp.	81	100	92	50	83	44	91	85	60	80	1
*Acidovorax* spp.	69	100	92	33	80	67	100	90	60	80	2
*Mycobacterium* spp.	75	67	75	67	83	56	100	85	80	80	1
*Variovorax* spp.	44	100	92	50	80	56	100	95	100	80	3
*Brevundimonas* spp.	69	100	25	33	76	100	100	100	40	76	4
*Phenylobacterium* spp.	81	100	83	50	59	100	91	90	40	75	2
*Bosea* spp.	50	100	50	67	78	100	100	75	40	74	3
*Ramlibacter* spp.	44	100	75	50	80	44	100	85	60	73	2
*Hydrogenophaga* spp.	44	100	92	33	76	33	100	85	60	72	2
*Achromobacter* spp.	25	100	67	33	80	67	73	85	100	70	2
*Caulobacter* spp.	63	100	75	33	54	100	91	85	60	70	2
*Paracoccus* spp.	50	100	0	50	67	100	100	70	100	67	4
*Afipia* spp.	69	100	58	50	70	100	91	20	40	64	2
*Rhodoferax* spp.	13	100	67	17	65	22	100	90	60	61	2
*Rhodopseudomonas* spp.	56	100	75	50	61	100	64	35	60	61	2
*Microvirga* spp.	31	100	0	67	74	100	100	40	80	61	3
*Nitrospira* spp.	75	100	100	17	52	0	100	55	60	61	3
*Hyphomicrobium* spp.	31	100	8	17	80	100	100	40	40	61	3
*Rhodoplanes* spp.	31	100	58	67	70	100	91	25	40	61	2
*Comamonas* spp.	31	100	83	17	57	22	82	90	60	61	1
*Polaromonas* spp.	19	100	58	17	70	22	82	80	40	59	1
*Methylorubrum* spp.	31	83	0	50	63	100	100	55	40	58	2
*Flavobacterium* spp.	38	33	67	50	50	11	91	90	60	56	0
*Noviherbaspirillum* spp.	19	100	67	17	65	33	82	50	60	55	1
*Erythrobacter* spp.	94	0	0	17	33	100	100	90	40	55	2
*Aromatoleum* spp.	50	100	92	50	48	44	73	45	20	55	1
*Thauera* spp.	31	100	75	50	57	11	91	45	40	54	1
*Nitrosomonas* spp.	50	100	25	17	52	0	100	70	40	52	2
*Legionella* spp.	69	50	8	17	65	11	82	50	60	52	0
*Paenibacillus* spp.	50	100	0	50	57	44	55	65	60	52	1
*Curvibacter* spp.	19	67	67	17	46	44	100	70	40	52	1
*Sphingorhabdus* spp.	88	0	58	0	52	11	36	80	20	51	0
*Lysobacter* spp.	31	100	0	17	70	33	18	70	60	51	1

Members of the *Sphingomonas* genera are Gram‐negative bacteria isolated from many different land and water habitats thanks to their ability to survive at low nutrient concentrations. More recently, the genus has been subdivided into different genera, in which we can find two other prevalent microbial groups reported above, i.e. *Novosphingobium* and *Sphingobium* (Takeuchi *et al*., 2001). Conversely, members of the *Bradyrhizobium* and *Mesorhizobium* genera are Gram‐negative nitrogen‐fixing bacteria that occur either as free‐living soil bacteria or that are found in symbiotic interaction within the roots of leguminous plants (Lorite *et al*., 2018). Similarly, members of the *Paraburkholderia* genus are also nitrogen‐fixing bacteria correlated with plant growth promotion, while members of the related genus *Burkholderia* can also be pathogens for humans, being the case for several species of the *Pseudomonas* genus. However, since these latter microorganisms are ubiquitously distributed in drinking water, they are probably not harmful to humans. Nevertheless, the presence of opportunistic pathogens in rainwater and tap water storage systems has already been discussed, showing the natural occurrences of *Pseudomonas aeruginosa*, *Legionella* spp. and *Mycobacterium* spp. (Zhang *et al*., 2021). Thus, it would be of great interest to unveil the identified taxa genomic capability so as to assess and understand possible interactions with humans upon ingesting water containing such microbes.

Recently, high‐throughput molecular analyses of microbiomes have been used as a tool to monitor the wellbeing of aquatic environments, involving cultural‐independent analyses such as metagenomics, metatranscriptomics, metaproteomics and metabolomics (Michán *et al*., 2021). In the frame of this work, water microbiome profiling revealed a conserved microbial core represented by mostly unclassified and uncharacterized bacteria, which highlights a dearth of knowledge from a genomic and functionality perspective. Thus, microbiomes of waters should be investigated through culturomics experiments to gain access to such novel microbial species. In addition, since many microorganisms cannot be cultivated using standard procedures, deep metagenome sequencing can be performed to reconstruct unknown bacterial genomes.

### Investigating the impact of tap water on the human microbiome

Recent literature has supported the notion that ingestion of foods populated by a specific microbiota facilitates transmission and subsequent colonization of these microorganisms in the human gut (Hehemann *et al*., 2010; Makki *et al*., 2018; Milani *et al*., 2019). Since every person consumes about 2 L of water per day, we explored the influence of water consumption on shaping the composition of the human gut microbiota. Therefore, bacterial DNA sequences collected from the 115 drinking water samples belonging to P02–P10 were subjected to genome reconstruction to gather chromosomal fragments of unknown bacterial taxa. In total, 2.9 gb of DNA sequences belonging to unknown bacteria were assembled in this manner. Nonetheless, we decided to investigate only those bacteria identified with the highest prevalence in water samples of P1 and the other projects (Table 2) represented by 622 mb of as yet unclassified members of the genera *Bradyrhizobium*, *Burkholderia*, *Mesorhizobium*, *Novosphingobium*, *Paraburkholderia*, *Pseudomonas*, *Reyranella*, *Sphingobium*, *Sphingomonas* and *Sphingopyxis*.

**Fig. 3:**
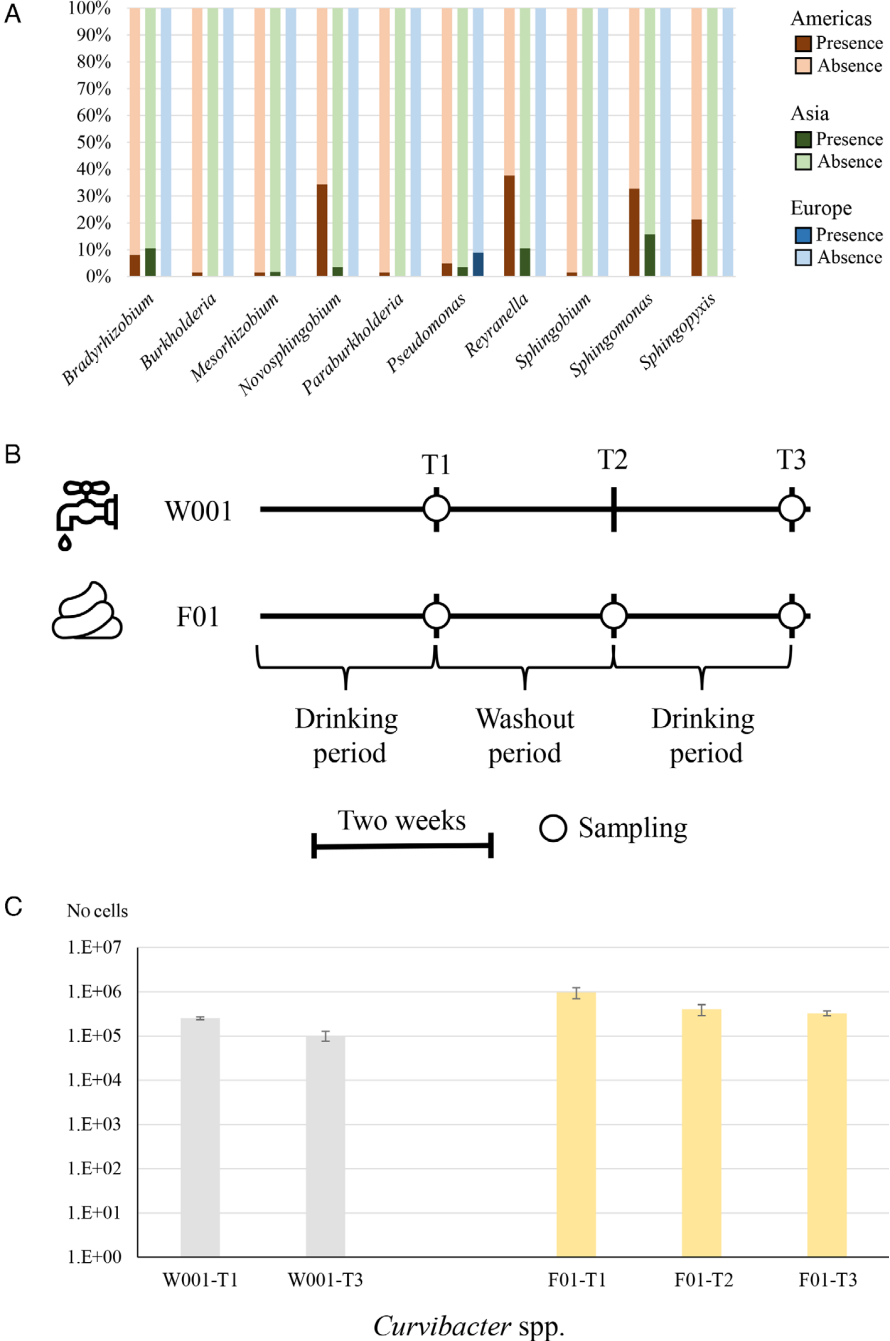
Investigation of the colonization of drinking water bacteria in humans. Panel A shows the percentage of human faecal samples in which the 10 core microorganisms associated with drinking water were identified through read mapping. Panel B represents a schematic representation of the pilot experiment with drinking and sampling periods (T1, T2 and T3). Panel C illustrates the qPCR data of *Curvibacter* spp. in tap water (W001‐T1 and W001‐T3) and faecal samples of the subject (F01‐T1, F01‐T2 and F01‐T3).

To trace the presence of the assembled microbial genomes, we included in this study shotgun metagenomic data of 196 human faecal samples retrieved from the NCBI repository (Table S2). Selection of faecal samples was performed so as to equally cover countries from which the drinking water was collected (Table S2). Then, the total amount of microbial DNA retrieved from the latter samples was used to investigate the presence of bacterial dark matter reconstructed through shotgun metagenomic assemblies. DNA mapping was performed with high sensitivity and high specificity (see Experimental procedures), identifying DNA of the reconstructed bacteria from the water microbiome in 46 of the analysed human faecal samples (Fig. 3). DNA sequences corresponding to unclassified bacteria were distributed across the three analysed continents, with a higher predominance of sequences belonging to *Sphingomonas* and *Reyranella*, both identified in 43 faecal samples, followed by *Novosphingobium* in 37 faecal samples. Following the latter microorganisms, the DNA of *Sphingopyxis*, *Pseudomonas* and *Bradyrhizobium* was identified in 27–25 samples, while the remaining four genera were identified in 16–14 faecal samples (Fig. 3). Altogether, these data indicate that the DNA of microorganisms belonging to the core microbiota of drinking water samples can also be detected in human faecal samples. Overall, these findings suggest that such tap water‐associated microorganisms contribute to the human gut microbiota composition. A further interesting aspect awaiting to be investigated will be how many of these water‐associated microorganisms found in the gut of humans were transmitted through direct ingestion of water or to the extensive use of tap water in watering plants for consumption.

### Transmission of microorganisms from tap water to the human gut

The *in silico*‐based findings indicate the occurrence of microbial DNA belonging to the core tap water microbiota in various human faecal microbiomes. This prompted us to investigate this potential novel route for shaping the human gut microbiota. Thus, we examined the gut microbiota composition, together with the corresponding tap water microbiota, of a subject who daily consumed tap water for the past 3 years. Specifically, tap water consumed by the subject had been profiled in the context of this work (corresponding to W001) and subjected to metagenome assembly of its microbial DNA content. Assembled bacterial DNA resulted in 104 kb distributed in 40 contigs, reflecting portions of chromosomes of those microorganisms present in relatively high abundance within the sample. Notably, assembled contigs, which taxonomically were predicted to belong to putative unknown species of the genus *Bradyrhizobium*, *Curvibacter* and *Sphingobium*, were used to design primer pairs for quantitative real‐time PCR (qPCR) investigation.

Faecal sample collection was accomplished through three time points aimed at investigating the microbiota of the subject during the consumption of W001 (T1 and T3) and after a period of washout of 2 weeks, during which the subject drank only bottled water (T2) (Fig. 3). Notably, bottled water microbiota was analysed, revealing a negligible amount of microbial DNA, thus corroborating the microbiological sterility of the administered water between T1 and T2. Due to the high CFU ml^−1^ identified in sample W001 at T1 (5.3 × 10^6^), an additional (FC) assay was performed at T3 of the same tap water highlighting a consistent quantification of microbial cells (4.8 × 10^6^). Remarkably, employing the *Bradyrhizobium* and *Sphingobium* strain‐specific primers, qPCR assays on the faecal samples resulted in quantifying DNA below the detection limit. Instead, *Curvibacter* DNA was identified at each time point, indicating apparent colonization of this *Curvibacter* species in the subject's gut as it was detected even after 2 weeks following the start of the washout period (Fig. 3). Notably, even if these data have been obtained from a single individual, they suggest that some microbial species residing in tap water can survive to the human colonic tract and colonize and persist in the gut of their human host. A clinical trial involving a substantial number of subjects will need to be executed to validate these findings.

## Conclusions

Tap water is considered a food, providing essential elements to our body, which are vital for our lives. However, tap water can also be a reservoir of microorganisms that, once ingested, may colonize our intestine, influence the gut microbiota and be responsible for different metabolic activities associated with human health. In the current study, we were interested in assessing the notion that water is not only crucial for our nutritional and physiological requirements but may also be important as a delivery vehicle of microorganisms to the gut. Notably, and in contrast to most consumed foods, the microbial community composition of tap water is not very well studied (Sala‐Comorera *et al*., 2020). Here, we clearly show that a large part of the microorganisms present in water is represented by as yet to be characterized bacteria, thus representing constituting a lot of microbial dark matter. These findings should prompt dedicated investigations on these bacteria, aimed at isolation, cultivation and subsequent dissection of their biological features. In fact, as outlined in our study, a large number of these putative novel bacterial taxa, which make up part of the core tap water microbiota, are also identified as part of the human faecal microbiota representing various different metagenomes and geographical regions. Thus, this co‐sharing scenario of members of the core tap water microbiota and the human gut microbiota may impact human health through modulation of the gut microbiota. This may therefore represent an intriguing and novel scenario that warrants further careful exploration. Here, our findings indicate that horizontal transmission and subsequent colonization of microorganisms from tap water to the human gut is possible. However, a clinical trial encompassing a more extensive set of individuals drinking tap water encompassing different microbiota and involving the isolation of the microorganisms using culturomics approaches needs to be performed in order to corroborate our data.

## Experimental procedures

### Tap water samples and sampling conditions

Sixteen tap water samples, including public fountains and household taps, were randomly selected from different locations and distribution systems encompassing various parts of Parma town and its territory. To ensure that the collected samples are representative of consumed water, at least 5 L of water was directly collected from the tap, keeping at a safe distance from the faucet and letting some flow down before directly flushing the water into sterile bottles to minimize any contaminations. Water samples were transported to the laboratory and kept at 4°C for further analysis.

### Microbial DNA extraction

For bacterial DNA extraction, 5 L of a given water sample was filtered through 0.45 μm pore size hydrophilic mixed cellulose esters (Pall Corporation, Port Washington, NY, USA). Filters were placed in standard Petri dishes and were cut into small pieces to ensure total sterility. DNA was extracted from the filters using the ZymoBIOMICS DNA Miniprep Kit (Zymo Research, D4300) following the manufacturer's instructions. Then, each tap water sample's DNA concentration and purity were investigated by employing a Picodrop microtiter Spectrophotometer (Picodrop, Hinxton, UK).

### Shallow shotgun sequencing

According to the manufacturer's instructions, DNA library preparation was performed using the Nextera XT DNA sample preparation kit (Illumina, San Diego, CA, USA). First, 1 ng input DNA from each sample was used for the library preparation, which underwent fragmentation, adapter ligation and amplification. Then, Illumina libraries were pooled equimolarly, denatured and diluted to a concentration of 1.5 pM. Next, DNA sequencing was performed on a MiSeq instrument (Illumina) using a 2× 250 bp Output sequencing Kit together with a deliberate spike‐in of 1% PhiX control library.

### Short read taxonomic classification

Sequenced paired‐end reads of each water sample were subjected to a filtering step removing low‐quality reads (minimum mean quality score 20, window size 5, quality threshold 25 and minimum length 100) using the fastq‐mcf script (https://github.com/ExpressionAnalysis/ea-utils/blob/wiki/FastqMcf.md) to analyse high‐quality sequenced data only. Then, an additional filtering step was performed to remove possible contaminating human DNA sequences from each sample through reads mapping employing the BWA aligner (Li and Durbin, 2009). Filtered reads were then collected and taxonomically classified through the METAnnotatorX2 pipeline (Milani *et al*., 2021), using a set of databases of reference genomes whose taxonomy was previously validated to maximize the accuracy of homology‐based taxonomic classification of reads (Milani *et al*., 2021).

### Metagenome assembly

Filtered reads were subjected to whole metagenome assembly using Spades v3.15 (Wedemeyer *et al*., 2017) with default parameters and the metagenomic flag option (‐meta) together with k‐mer sizes of 21, 33, 55 and 77. As mentioned above, for the short reads, reconstructed contig sequences were taxonomically classified based on their sequence identity using megablast against the same RefSeq database (Chen *et al*., 2015). ORFs of each assembled genome were predicted with Prodigal (Hyatt *et al*., 2010) and annotated utilizing the MEGAnnotator pipeline (Lugli *et al*., 2016). In all, the METAnnotatorX2 pipeline was employed for various purposes, from read filtering to taxonomic classification of the assembled contigs (Milani *et al*., 2018, 2021).

### Flow cytometry analysis

The samples for FC were collected in sterilized screw tap tubes (Sarstedt) and were transported to the laboratory within 1 h of collection, temporarily stored at 4°C, and measured within a few hours after collection. Then, 1 ml of water sample was stained with 1 μl ml^−1^ SYBR Green I (1:100 dilution in DMSO; Molecular Probes, Eugene, OR, USA) and incubated in the dark for 15 min before measurement. Count experiments were performed using an Attune NxT flow cytometer (Thermo Fisher Scientific, Waltham, MA, USA) equipped with a blue laser set at 50 mW and tuned to an excitation wavelength of 488 nm. Multiparametric analyses were performed on scattering signals, i.e. forward scatter and side scatter, and SYBR Green I fluorescence was detected on the FL1 channel. The detection limit was determined experimentally by filtering one aliquot of water sample and one of Attune Focusing Fluid 1× through 0.20 μm pore size hydrophilic mixed cellulose esters (Pall Corporation). Then 1 ml of each sample was stained with 1 μl ml^−1^ SYBR Green I as mentioned above. Cell debris was excluded from the acquisition analysis by a sample‐specific FL1 threshold, and collected data were statistically analysed with Attune NxT flow cytometer software.

### DNA mapping

Microbial DNA retrieved from 197 human faecal samples was aligned to the reconstructed chromosomal portions of unknown water bacteria to evaluate the presence of water microorganisms in the gut of humans. The Bowtie2 program was used to align the DNA sequences through multiple‐hit mapping and a ‘very sensitive’ policy (Langdon, 2015). The mapping was performed using a minimum score threshold function (–score‐min C,‐13,0) to limit reads of arbitrary length to one mismatch and retain those matches with at least 99% full‐length identity. The SAMtools software package (Danecek *et al*., 2021) was then used to count the mapped reads among each bacterial taxon, rejecting hits with less than 10 reads to achieve a consistent output.

### Experimental design

The experiment involves a healthy adult male who daily drank tap water corresponding to sample W001 for the last three years. The objective was to collect faecal samples before and after the two weeks of the washout period. The first faecal sample collection was performed before the washout (F01‐T1) to identify bacteria introduced by the consumption of tap water. Then, we collected faecal samples at T2 and T3 to cover the end of the washout and the restoration of W001 administration. Faecal samples were stored at −80°C until use. Concomitantly, W001 was collected at T1 and T3 using the procedure reported above.

### Quantitative real‐time PCR

The abundance of microorganisms identified in W001 was evaluated through qPCR in faecal sample F01. The DNA of F01 was extracted and diluted at a concentration of 10 ng. The presence of *Bradyrhizobium* spp., *Sphingobium* spp. and *Curvibacter* spp. DNA was evaluated using q‐PCR with primer pair (5′‐TGCGGTCACTCATCTTAGCT‐3′/5′‐GAGAACGCACGATCACCTTC‐3′), (5′‐CTGAACTGTTCGATCGGCTG‐3′/5′‐GCCATCGACCTCCTTATCCA‐3′) and (5′‐AGACCAGCTACAGATCGTCG‐3′/5′‐TACACATGCAAGTCGAACGG‐3′) respectively. In detail, qPCR was performed using SoFast EvaGreen Supermix (Bio‐Rad) on a CFX96 system (BioRad, CA, USA) following previously described protocols (Milani *et al*., 2015). Each PCR reaction mix contained the following: 12.5 μl 2× SYBR SuperMix Green (BioRad), 5 μl of DNA at a concentration of 10 ng μl^−1^, each of the forward and reverse primers at 0.5 μM, and nuclease‐free water was added to obtain a final volume of 20 μl.

### Statistical analysis

Bacterial abundance at the species level was validated by ANOVA analysis. Furthermore, PERMANOVA analysis was performed using 1000 permutations to estimate *p*‐values of differences among water samples in PCoA analyses. Statistical analyses were performed by using OriginPro graphing and analysis 2021.

## Author Contributions

G.A.L. performed bioinformatics analyses and wrote the manuscript; G.L. performed the *in vitro* analyses and edited the manuscript; G.A. and C.T. validated the *in vitro* analyses; L.M. and F.F. validated the bioinformatics analyses; F.T., C.M., and D.v.S. supervised the project and edited the manuscript; M.V. designed the study and edited the manuscript.

## Ethics Statement

Faecal experimental procedures and protocols involving human faecal samples were approved by the Ethics Committee of Parma University. Signed informed consents were obtained from the individuals enrolled in this study.

## Supporting information


**Fig. S1.** Absolute abundance of the microbial composition of 16 drinking water samples collected from different locations of the Parma district and delivered by the city water supply system. Due to the high CFU ml^−1^ of sample W001, its histogram is reported with a different CFU ml^−1^ scale to appreciate the absolute abundance of the microbial community in the other 15 water samples.

**Table S1.** Shallow shotgun sequencing filtering data
**Table S2.** Public shotgun sequencing metadata
**Table S3.** Biodiversity between water projects.

## Data Availability

Shotgun metagenomics data are accessible through SRA study accession number PRJNA806724.
