# Supplementary figures and images for: Tap water as a natural vehicle for microorganisms shaping the human gut microbiome
Source: Environ Microbiol. 2022 Apr 7;24(9):3912–23. doi: 10.1111/1462-2920.15988 (PMC9790288; doi:10.1111/1462-2920.15988)

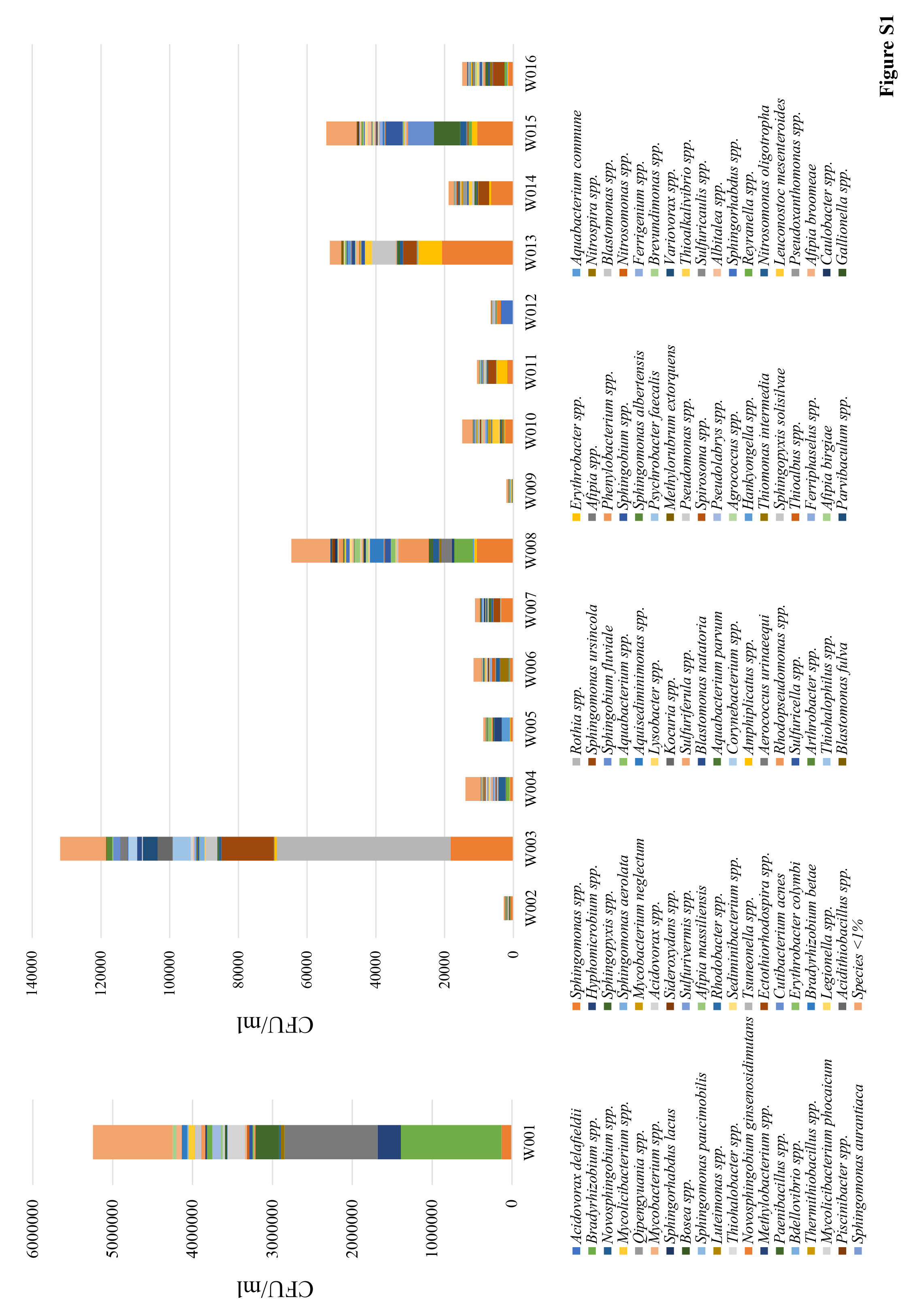

Supplement: Supplementary file 1 — Fig. S1. Absolute abundance of the microbial composition of 16 drinking water samples collected from different locations of the Parma district and delivered by the city water supply system. Due to the high CFU ml−1 of sample W001, its histogram is reported with a different CFU ml−1 scale to appreciate the absolute abundance of the microbial community in the other 15 water samples. [file EMI-24-3912-s001.tif]
